# Focal Atrial Tachycardia in a Patient With Surgically Corrected Tetralogy of Fallot

**DOI:** 10.14740/cr316w

**Published:** 2014-02-27

**Authors:** Mario Gonzalez, Ricardo Castillo, Asma Syed

**Affiliations:** a Brookdale Hospital and Medical Center, 1 Brookdale Plaza, Brooklyn, NY 11212, USA

**Keywords:** Congenital heart disease, Arrhythmias, Atrial tachycardias, Post surgical scar

## Abstract

Tetralogy of Fallot (TOF) is a cyanotic congenital heart disease which, without corrective surgery, has a poor prognosis. These patients have an increased incidence of arrhythmias both supraventricular and ventricular post surgical correction. The supraventricular arrhythmias are usually related to the scar tissue at the surgical repair site. We present a case of a young male patient status post TOF repair who presented with a supraventricular tachycardia which was found to be unrelated to his surgical scar.

## Introduction

Congenital heart disease patients have a high prevalence of atrial tachycardias (ATs) especially after corrective surgery [1]. Macroreentrant AT is the most common mechanism of AT in this patient population. Focal ATs are rarely reported in these patients. A study looking at focal AT in these patients demonstrated that the site of origin for these tachycardias is still usually near the surgical scar [2]. Our patient appeared to clinically have a macroreentrant tachycardia (based on the 12-lead electrocardiogram (EKG)); however, mapping demonstrated it to be a focal atrial tachycardia (FAT) unrelated to his surgical scar.

## Case Report

**Figure 1:**
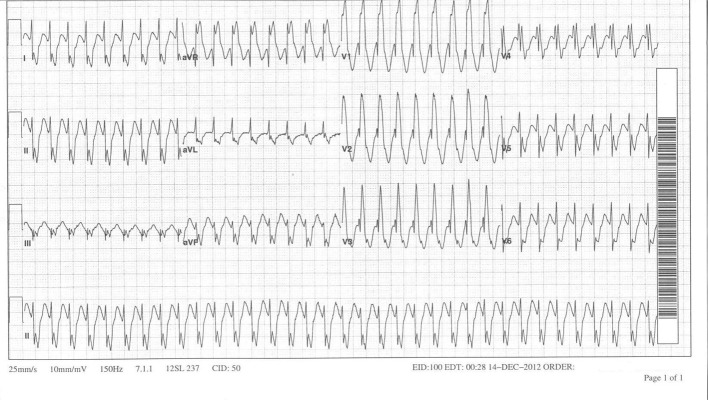
Initial tachycardia - wide complex tachycardia with an RBBB pattern and possible retrograde P waves.

**Figure 2:**
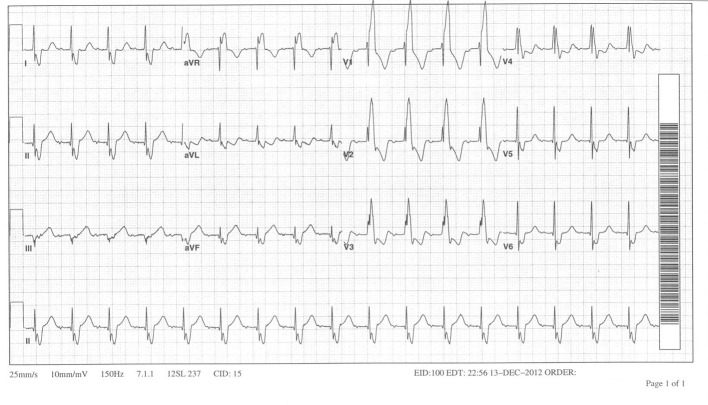
After administration of IV adenosine and diltiazem demonstrating slower tachycardia with an RBBB pattern and possible 2:1 conduction.

A 39-year-old male patient with a past medical history significant of tetralogy of Fallot (TOF) s/p surgical repair as a child, presented to the emergency room with complaints of palpitations. On presentation, his vitals included a heart rate of 206 bpm and a blood pressure of 115/65 mmHg. His 12-lead EKG (Fig. 1) demonstrated a tachycardia with a right bundle branch block (RBBB) pattern and a possible retrograde P wave. The patient was given IV adenosine and diltiazem with some resolution of his symptoms. His repeat 12-lead EKG revealed a persistent RBBB with a heart rate around 100 bpm and a possible 2:1 tachycardia (Fig. 2). The P wave was noted to be negative in V1, positive in AVR and AVL, negative in the precordial leads and biphasic in the inferior leads. Patient subsequently underwent a transesophageal echocardiogram which was negative for a left atrial thrombus followed by an electrophysiology study and radiofrequency ablation.

**Figure 3:**
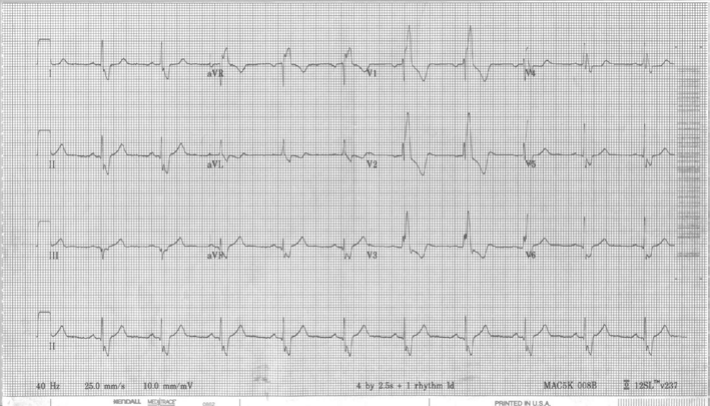
Post procedure EKG demonstrating normal sinus rhythm with an RBBB pattern.

During the electrophysiology study, patient was noted to be in a 2:1 arrhythmia with a cycle length 303 ms and earliest activation on the right-sided electrodes of the coronary sinus catheter. Entrainment mapping was attempted but was not successful. Detail 3D mapping with Biosense Webster’s CARTO mapping system was performed which demonstrated the tachycardia to be a right-sided FAT. The earliest activation of the tachycardia appeared to be at the lateral free wall of the atrium by the crista terminalis. Complex electrograms were noted at the site of the earliest activation. Radiofrequency energy was applied at the site of the earliest activation which resulted in termination of the tachycardia. Despite repeat pacing maneuvers and isoproterenol infusion, tachycardia was not re-induced. Post procedure and on subsequent follow-ups, patient’s 12-lead EKG (Fig. 3) demonstrated normal sinus rhythm with an RBBB.

## Discussion

TOF is one of the most common cyanotic congenital heart diseases (CHDs) [3]. Patients post surgical repair are at risk for scar-related arrhythmias [4]. Macroreentrant AT has been found to be the most common mechanism of tachycardia in these patients [1]. FAT is rarely reported in post surgical patients [1]. For patients with CHD, arrhythmias tend to be an active issue by the time they reach adolescence or adulthood [5]. The arrhythmias are usually divided into two causes, one secondary to structural malformation of the CHD itself and the second, most common due to unique substrate created by the surgical scar. There is a high prevalence of sustained atrial arrhythmias as well as ventricular arrhythmias in patients post surgical repair for TOF [6]. The incidence of reentrant atrial arrhythmias appears to be more of a right-sided arrhythmia in these patients. Also there might be multiple intra-atrial reentrant circuits present due to the surgical scars [7]. In a retrospective study, the incidence of atrial arrhythmias was linked to a two- to threefold increase risk of developing congestive heart failure [8]. FAT in CHD patients is an uncommon finding [2]. There have been a few case reports describing the presence of FAT in post surgical CHD patients; however, most of those cases documented the site of earliest activation to be in close proximity to the surgically created barriers (scar tissue, suture lines, and so on) [9, 10]. In one study, FATs were noted to be not only close to surgical scar sites but rarely also from sites common in patients with normal hearts [2]. FAT in normal hearts usually arises from the crista terminalis in the right atrium and pulmonary veins in the left atrium [11]. Our patient presents a common arrhythmia in an uncommon situation given his significant cardiac history.

P wave morphology algorithm is used to predict the focal site of the AT [12].

In patients who have had surgical procedures done, the P wave morphology tends to be less useful. In our patient, the P wave morphology was more consistent with an AT arising from the tricuspid annulus; however, mapping demonstrated the tachycardia to be arising from the lateral free wall of the right atrium.
